# Skeletal Muscle Mass as a Mortality Predictor among Nonagenarians and Centenarians: A Prospective Cohort Study

**DOI:** 10.1038/s41598-019-38893-0

**Published:** 2019-02-20

**Authors:** Hui Wang, Shan Hai, Yixin Liu, Ying Liu, Birong Dong

**Affiliations:** 1 Department of Geriatrics and National Clinical Research Center for Geriatrics, West China Hospital of Sichuan University, Chengdu, China; 2 Center of Gerontology and Geriatrics, West China Hospital of Sichuan University, Chengdu, China

## Abstract

This study aimed to evaluate the association between skeletal muscle mass and long-term all-cause mortality among nonagenarians and centenarians in China. We used data from the Project of Longevity and Aging in Dujiangyan (PLAD). A total of 738 community-dwelling people aged ≥ 90 years (mean age of 93.5 ± 3.2 years) were analyzed in this study. The appendicular skeletal muscle mass (ASM) was estimated using a previously validated anthropometric equation. The information on the survival status was requested from the local government registries during the 4 year follow-up period following the baseline investigation. The mean muscle mass index (SMI) was 6.11 ± 0.53 kg/m^2^ in men and 4.00 ± 0.63 kg/m^2^ in women, respectively. Low muscle mass was associated with a higher risk of death (hazard ratio [HR] 1.54; (95% confidence interval [CI]:1.10–2.16) in women; however, no significant association was found in men. Disability in activities of daily living (ADL) (HR = 1.73; 95% CI: 1.13–2.63) in men and women and cognitive impairment (HR = 1.49; 95% CI: 1.05–2.13) in men were also associated with increased all-cause mortality. In conclusion, low muscle mass were predictors of long-term mortality in nonagenarian and centenarian women.

## Introduction

The number of advanced aging individuals is rapidly increasing worldwide, and the percentage of people aged 65 years and over in China is projected to increase from 7.1% to 14.9% between 2000 and 2030^1^. The population group aged 90 years and over is the fastest-growing population in China (from 971,227 in 2000 to 1,984,220 in 2010^2^), and this increase has become a public health concern. Previous studies have prospectively evaluated the predictors of mortality in nonagenarians or centenarians. In general, geriatric syndromes, such as disability, mobility limitation, cognitive impairment, and poor physical performance, were positively associated with mortality^3–9^. However, low muscle mass, another important geriatric syndrome, did not receive ample attention.

Rosenberg first focused on the decline in muscle mass in elderly population and described the age-related loss of muscle mass as Sarcopenia^10^. The previous studies identified that muscle mass was a primary influencing factor for the changes in function, and muscle mass deficiency was associated with poor health outcomes, including functional impairments, physical disability, and mortality in elderly individuals^11–13^. Cheung and colleagues reported that the appendicular lean mass (ALM) alone and ALM adjusted for body mass index could predict mortality in the National Health and Nutrition Examination Survey 1999–2004^14^. Visser *et al*. reported that lower muscle mass and muscle strength are both associated with mobility limitations in elderly individuals in the Health ABC study^15^. Recently, Weng and colleagues demonstrated that mid-arm circumference (MAC) and calf circumference (CC), the surrogate markers of muscle mass, were independent predictors for 7-year mortality^16^. However, all these studies were not designed for the oldest-old people who were generally considered as a special group of “escapers” or being resilient to common diseases due to several physiological and pathological characteristics. It remains unclear whether low muscle mass is a predictor of mortality in nonagenarians and centenarians with more frailty and disability. Therefore, the aim of this study was to assess the muscle mass in nonagenarians and centenarians by using an anthropometric equation, and investigate the association between muscle mass deficiency and all-cause mortality in the oldest-old population.

## Methods

### Participants

The present study is a secondary analysis of a prospective population-based cohort of nonagenarians and centenarians in 2005, the Project of Longevity and Aging in Dujiangyan (PLAD), which aimed to investigate the relationships among environment, genes, lifestyle, cognitive function, longevity, and age-related chronic conditions, and the vital information was collected in 2009 from the local government registers. In the summer of 2005, a total of 1115 residents aged ≥ 90 years in Dujiangyan were screened, and researchers surveyed 870 residents of the population, with a remarkable “capture rate” (78%). In the summer of 2009, after 4 years of follow-up, mortality data were requested from local government registries, relatives, or neighbors. Fifty-five participants (19 men and 36 women) were excluded because of the lack of vital status data due to the loss of contact and inability of the local government to complete the search within the given time. A total of 90 participants (27 men and 63 women) were excluded due to missing data on half arm span or weight. The analysis was conducted based on the data from 738 people (238 men and 500 women). Data collection was approved by the Research Ethics Committee of the Sichuan University. All participants (or their legal proxies) signed a written informed consent. All methods in this study were performed in accordance with relevant guidelines and regulations.

### Assessment of muscle mass

Body weight was measured using a digital floor scale to the nearest 0.1 kg. We used arm span to surrogate height to avoid underestimation, because 100 participants (13.6%) had vertebral compression and kyphosis^17^. Half arm span, the distance from the middle of the sternal notch to the tip of the middle finger, was measured using the tapeline to the nearest 0.1 cm, and height was calculated by doubling the measurement of the half arm span. Muscle mass was estimated by the appendicular skeletal muscle mass (ASM) using an equation that was a previously validated in a Chinese population: ASM = 0.193*body weight + 0.107*height-4.157*sex-0.037*age-2.631^18^. The body weight, height, and age were measured in kilograms, centimeters, and years, respectively. For sex, the value 1 represented men, and the value 2 represented women. The muscle mass index (SMI) was calculated using the ASM divided by the square of the height in meters (SMI = ASM/height^2^). Because no consensus cutoff point has been adopted yet for elderly individuals aged ≥ 90 years, participants were categorized to low muscle mass group if their SMI was 1 SD below the mean of the study sample. The cutoff point that was adjusted for gender was <5.58 kg/m^2^ for men and <3.38 kg/m^2^ for women.

### Assessment of covariates

The following covariates were collected by trained interviewers: age, gender, education (any education or illiteracy), alcohol status (current, former, or not), and smoking status (current, former, or not). Information on geriatric conditions including hearing problems, vision problems, fall (in the past year), and fracture (any kind) was collected by trained personnel through face-to-face interviews. The following self-reported chronic diseases that may be related with mortality were also assessed: hypertension, chronic heart disease, cerebrovascular disease, peripheral vascular disease, Parkinsonism, diabetes mellitus, respiratory diseases, gastrointestinal diseases, chronic renal disease, prostatic diseases, osteoarthritis and cancer. Disability was evaluated with self-reported activities of daily living (ADL) by using the Katz Index with the six basic ADLs (bathing, dressing, toileting, transferring, continence, and feeding). “Not disabled” was defined as the independent performance of all items, “moderately disabled” as the dependent performance of one or two items, and “severely disabled” as the dependent performance of three or more items in accordance with the definitions given in the Katz’ paper^19^. Cognitive status was measured by using the Mini-Mental State Examination (MMSE). The individuals were categorized based on the following: severe cognitive impairment (score: ≤ 17), mild cognitive impairment (MCI) (scores: 18–23), and normal (score: ≥ 24)^20^. In addition, we measured MAC and CC using a millimeter graded tape to the nearest 0.1 cm. Venous blood samples were collected after an overnight fast to measure plasma glucose, plasma lipid, and serum albumin levels and other biochemical indicators.

### Survival status

The survival status was requested from local government registries and was confirmed by relatives or neighbors from summer 2005 (original date of the PLAD study) to summer of 2009. For the participants who died during the follow-up, the period from the baseline investigation to the date of death was recorded; for the individuals who survived during the study follow-up, the period from the baseline investigation to the end of the follow-up was recorded. Data on the cause of death were not available.

### Statistical analysis

Statistical analyses were performed by using SPSS version 18.0 for Windows (IBM Corporation, Armonk, NY). The continuous data were presented as the mean ± standard deviation (SD) if they were normally distributed; otherwise, they were presented as the median ± interquartile range. The categorical data were presented as absolute numbers and percentages (%) of the total. The difference between the low ASM group and the normal group were compared through independent t tests for continuous variables with a normal distribution, and the Mann–Whitney *U* test for continuous data with an abnormal distribution; and Pearson chi-square test or Fisher exact test (with the expected cell count of <5) for categorical variables. The Kaplan–Meier curves were plotted via the log-rank test to demonstrate the association between survival status and low muscle mass. The proportional hazard assumption has been checked by Schoenfeld residuals test (*p* > 0.05). The cox regression models were used to estimate the hazard ratios (HR) to identify the independent predictors of mortality, adjusting for potential confounders (age, sex, lifestyle, disability, and cognitive impairment). *P* value < 0.05 was considered statistically significant.

## Results

The mean age of the 738 participants was 93.5 ± 3.2 years (range: 90–105 years), and 43 were centenarians. The mean ages of the men and women were 93.2 ± 3.1 and 93.7 ± 3.3 years, respectively. Approximately 67.8% of the participants were women, 90.1% lived in the countryside, 79.6% were farmers before retirement, and 72.4% were illiterate. The percentages of the current alcohol drinkers and smokers were 25.8% and 44.1%, respectively. Men were reported to drink and smoke more than women (37.9% vs. 20% and 71.1% vs. 31.2%). The most prevalent chronic diseases were osteoarthritis (29.7%), respiratory diseases (14.4%), and gastrointestinal diseases (17.1%). The mean MMSE scores were 16.9 ± 5.9. About 51.6% of the participants suffered from severe cognitive impairment, 34.3% had mild cognitive impairment, and only 14.2% were normal according to the MMSE scores. The prevalence of disability was 33.1%, of which 3.3% accounted for severe impairment and 29.8% for moderate impairment.

The baseline characteristics of the participants are presented in Table 1. The mean muscle mass index (SMI) of the nonagenarians and centenarians was 6.11 ± 0.53 kg/m^2^ in men and 4.00 ± 0.63 kg/m^2^ in women, respectively. 32 men (13.4%) and 86 women (17.2%) were classified as participants with low muscle mass. The women with low muscle mass were older compared with the participants with normal muscle mass (94.7 ± 3.7 vs. 93.5 ± 3.2 years, p < 0.001); in addition, they had lower height, weight, BMI, MAC, and CC. The men with low muscle mass had lower weight and BMI, but similar MAC and CC; and the prevalence of osteoarthritis and visional problem was lower than the participants with normal muscle mass.

**Table 1:** Characteristics of Chinese Nonagenarians and Centenarians by muscle mass and gender.

	Men			Women		
	Low muscle mass	Normal muscle mass	*p*	Low muscle mass	Normal muscle mass	*p*
N	32	206		86	414	
Age (years)	94.0 ± 4.0	93.1 ± 2.9	0.211	94.7 ± 3.7	93.5 ± 3.2	0.001
Illiteracy (n, %)	11 (34.4)	76 (37.6)	0.724	78 (90.7)	369 (89.3)	0.709
Current alcohol drinker (n, %)			0.675			0.155
Current	8 (25.0)	80 (40.0)		19 (22.9)	79 (19.4)	
Former	10 (31.3)	63 (31.5)		11 (13.3)	48 (11.8)	
No	14 (43.8)	57 (28.5)		53 (63.9)	280 (68.8)	
Current smoker (n, %)			0.035			0.753
Current	23 (71.9)	144 (70.9)		23 (27.7)	130 (31.9)	
Former	2 (6.3)	40 (19.7)		19 (22.9)	86 (21.1)	
No	7 (21.9)	19 (9.4)		41 (49.4)	192 (47.1)	
Choronic diseases						
Osteoarthritis (n, %)	3 (12.0)	51 (33.3)	0.031	27 (42.9)	138 (43.7)	0.905
Gastrointestinal diseases (n, %)	5 (20.8)	26 (18.2)	0.778	22 (35.5)	73 (25.3)	0.115
Respiratory diseases (n, %)	5 (21.7.0)	28 (19.7)	0.783	14 (24.6)	59 (21.9)	0.655
Geriatric conditions						
ADL impairment (n, %)	9 (28.1)	45 (22.1)	0.448	37 (44.0)	152 (36.8)	0.213
Cognitive impairment (n, %)	17 (60.7)	131 (73.2)	0.174	64 (95.5)	339 (92.1)	0.326
Vision problems (n, %)	7 (25.0)	92 (52.3)	0.007	34 (47.9)	184 (51.0)	0.635
Hearing problems (n, %)	9 (33.3)	62 (34.8)	0.879	22 (30.6)	147 (39.5)	0.152
Falls (n, %)	14 (45.2)	102 (53.1)	0.640	34 (41.5)	174 (43.0)	0.903
Height (cm)	163.8 ± 10.1	162.9 ± 7.7	0.654	146.2 ± 6.3	151.2 ± 7.2	<0.001
Weight (kg)	37.4 ± 4.1	48.4 ± 6.9	<0.001	29.0 ± 2.5	40.5 ± 6.6	<0.001
BMI (kg/m^2^)	13.9 ± 0.8	18.2 ± 2.5	<0.001	13.6 ± 1.5	17.8 ± 2.9	<0.001
MAC (cm)	23.0 ± 6.0	23.7 ± 3.1	0.494	20.4 ± 3.0	23.4 ± 2.8	<0.001
CC (cm)	27.4 ± 3.5	28.4 ± 3.5	0.148	23.0 ± 2.8	25.4 ± 2.9	<0.001
ASM (kg)	14.5 ± 1.8	16.5 ± 1.8	<0.001	6.8 ± 0.9	9.6 ± 1.6	<0.001
SMI (kg/m^2^)	5.4 ± 0.2	6.2 ± 0.5	<0.001	3.2 ± 0.2	4.2 ± 0.5	<0.001
Albumin (g/L)	41.4 ± 7.7	42.6 ± 3.1	0.396	42.5 ± 2.9	43.1 ± 3.7	0.178

Mean and standard deviation are shown for continuous variables, proportions as percent are shown for categorical variables.

Using Pearson Chi-Square tests or Fisher’s exact test (with the expected cell count of <5) for categorical variables and independent t test for continuous variables, *p* < 0.05 was considered to be statistically significant.

MAC: mid arm circumference, CC: calf circumference, ADL: activities of daily living, ASM: appendicular skeletal muscle mass, SMI: skeletal muscle mass index.

**Figure 1:**
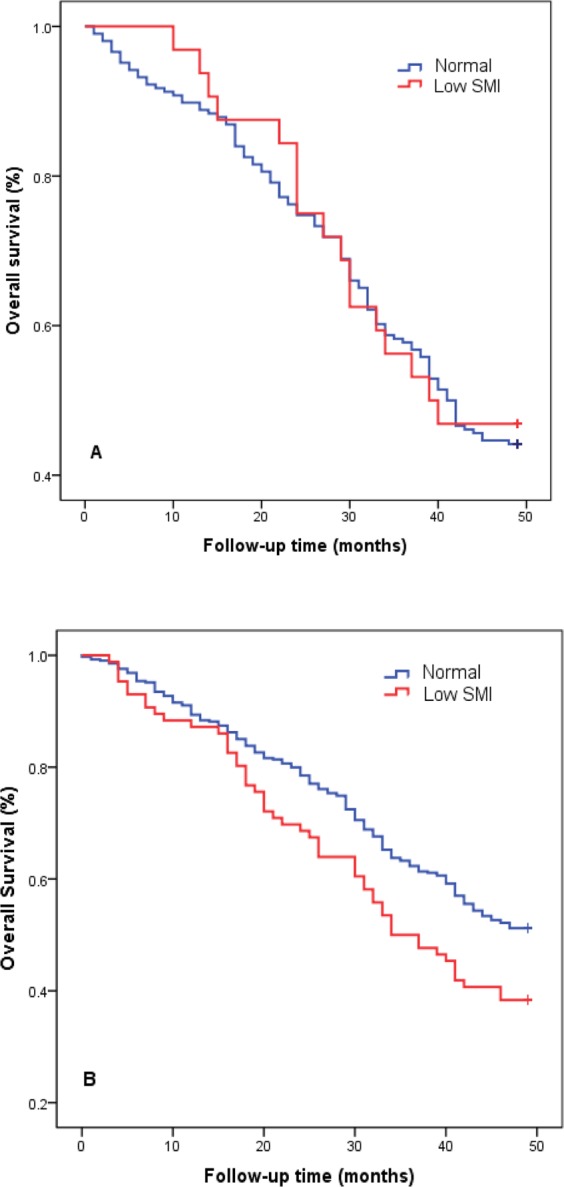
4-year survival probability of the oldest old according to appendicular skeletal muscle mass index. Survival curves were tested by log-rank test. Panel (A) male, *p* = 0.807; Panel (B) women, *p* = 0.016. Red line: low muscle mass index; Blue lines: normal muscle mass index.

A total of 387 participants (132 men and 255 women) died over the 4-year follow-up. The 4-year mortality was 52.4%, which is similar in men and women (55.5% vs. 51.0%, p = 0.257), and the mortality rates of the nonagenarians and centenarians were 52.2% and 55.8%, respectively. The women with low muscle mass had higher mortality compared with those with normal muscle mass (61.6% vs. 48.8%, p = 0.016), the difference was statistically significant; however, the men had no difference. The effect of low ASM on the 4-year mortality was tested using the Kaplan–Meier method. The survival curves of the participants with low or normal ASM are plotted in Fig. 1. Results of the log-rank test showed that the survival curves were significantly different (*P* < 0.001).

We calculated the HR using the Cox regression models that were adjusted for potential confounders. In an adjusted model, the women with low ASM had significantly increased HR for the 4-year mortality compared to those with normal muscle mass (HR = 1.54; 95% CI: 1.10–2.16) (Table 2); physical impairment, which affects the performance of ADL (HR = 1.54; 95% CI: 1.01–1.76) was also associated with increased mortality. In men, no significant difference in muscle mass was found between people who survived and those who deceased; however, disability (HR = 1.73; 95% CI: 1.13–2.63) and cognitive impairment (HR = 1.71; 95% CI: 1.07–2.73) significantly increased the 4-year mortality.

**Table 2:** Association between low muscle mass and mortality (4 year follow-up) according to Cox regression models adjusted for confounders.

Variables	Men			
	Unadjusted	Model 1	Model 2	Model 3
Low muscle mass	0.94 (0.56–1.56)	0.92 (0.55–1.54)	0.90 (0.54–1.52)	0.82 (0.45–1.47)
Age (years)		1.02 (0.97–1.08)	1.02 (0.97–1.08)	0.96 (0.89–1.04)
Smoker			1.02 (0.79–1.32)	1.13 (0.86–1.48)
Alcohol drinker			1.08 (0.88–1.34)	1.15 (0.91–1.45)
Cognitive impairment				1.71 (1.07–2.73)*
Disability				1.73 (1.13–2.63)*
**Variables**	**Women**			
	**Unadjusted**	**Model 1**	**Model 2**	**Model 3**
Low muscle mass	1.45 (1.07–1.96)*	1.36 (1.00–1.85)*	1.39 (1.01–1.90)*	1.54 (1.10–2.16)*
Age (years)		1.04 (1.00–1.08)*	1.05 (1.00–1.08)*	1.05 (1.00–1.09)*
Smoker			1.04 (0.90–1.20)	1.04 (0.89–1.22)
Alcohol drinker			1.05 (0.89–1.23)	1.04 (0.88–1.24)
Cognitive impairment				1.24 (0.71–2.18)
Disability				1.54 (1.01–1.76)*

Data are presented as hazard ratio (95% confidential intervals). *p < 0.05.

Model 1: adjusted for age.

Model 2: adjusted for age, smoking status, alcohol drinking status.

Model 3: adjusted for age, smoking status, alcohol drinking status, cognitive impairment, and disability.

## Discussion

To the best of our knowledge, this is the first study that focused on muscle mass as a predictor of long-term mortality in nonagenarians and centenarians. In this study, the muscle mass of the participants was 6.11 ± 0.53 kg/m^2^ in men and 4.00 ± 0.63 kg/m^2^ in women, respectively; and was associated with long-term mortality adjusted with other potential confounders for women.

Previous studies have addressed the decrease in ASM or SMI with age in various populations. In Hong Kong, a cohort study showed that the rate of decline in ASM is more rapid with increasing age, with a percentage loss −1.59% and −2.02% in men and women, respectively^21^. Another study on the Chinese community-dwelling elderly individuals is being conducted, and the authors showed that the SMI of the population aged ≥ 75 years was 7.09 ± 0.07 kg/m^2^ in men and 5.67 ± 0.07 kg/m^2^ in women, which were significantly lower than that of the preceding age group^22^. However, there lacks the reliable reference data for individuals older than 90 years. The mean SMI of the population was 6.11 ± 0.53 kg/m^2^ in men and 4.00 ± 0.63 kg/m^2^ in women in present study, respectively. If low muscle mass was defined according to the Asian Working Group for Sarcopenia (AWGS) algorithm^23^, only 17 participants (12 men and 5 women) met the cutoff value. So we identified that the long-lived populations were still not free of the decline of muscle mass and hypothesized that the low muscle mass might increase the mortality rate. Several studies identified that low muscle mass or various surrogate markers, including arm muscle area, MAC, CC, fat-free mass, and lean mass, were associated with an increased mortality in elderly^24–28^. However, studies on the association between muscle mass and mortality in the young-old population are not consistent; for example, the Invecchiare in Chianti (InChianti) Study reported that the muscle cross-sectional area of the calf was not related to the deaths in the population with a mean age of 74.5 years^29^. Our data also demonstrated that the low muscle mass only increased the female mortality in the oldest-old population; in men, the impact of the low muscle mass was less lethal compared to cognitive impairment and disability. The difference among gender might be due to using an equation in the limitation section and further research is needed.

Furthermore, several studies found that muscle strength or function began to decline even before muscle mass decrease and was better predictors of mortality^30–32^. Such discrepancy could be due to differences in study design, population studied, and method used for body composition measurement. As to extremely old individuals, the factors that predict mortality are generally not similar to those of the younger counterparts. The present study found that muscle mass is a predictor of long-term mortality in the oldest-old population. To the best of our knowledge, though previous studies on nonagenarians and centenarians have widely investigated functional and cognitive decline, only few studies evaluated the association between muscle mass or strength and mortality. Gueresi^33^ showed that mid-upper arm circumference, which is a helpful indicator of muscle mass, had prognostic significance for the survival of elderly Italian individuals aged 98 years and over. Another cohort study in Italy planned to investigate the body composition of nonagenarians. However, to date, only the baseline data from the survey were reported^34^. In addition, Taekema reported that poor handgrip strength predicts accelerated dependency in ADL and cognitive decline in oldest old^35^. Thus, more prospective studies should be carried out to confirm our findings.

Both cognitive impairment and disability are common in elderly patients^36^. Recently, a large cohort study, the Chinese Longitudinal Healthy Longevity Surveys (CLHLS), was conducted and reported that the prevalence rates of severe disability and dementia among nonagenarians were 12.4–15.4% and 27.6–41.2%, respectively; the prevalence rates among centenarians were 27.0–35.8% and 55.3–66.5%, respectively^37^. Our study identified the similar prevalence rate of severe cognitive impairment, that is, 50.7% among nonagenarians and 66.7% among centenarians. However, the prevalence of severe disability was significantly lower compared to the CLHLS study. In the present study, most elderly individuals with disability were classified under the group with moderate disability. The prevalence of severe disability was only 3.3% among nonagenarians and 2.2% among centenarians, respectively. The inconsistent findings were due to the discrepancy in the socioeconomic status of the two populations. In our study, approximately 90% of the participants were rural residents and 78% were farmers before retirement. In the CLHLS study, only 52.2–61.1% of the participants lived in the rural area^37^. Furthermore, the average annual disposable income among urban households was about 3.0 times higher than that of the rural households in 2005 and 2009, and the medical care status in rural area was also significantly poor. Thus, the elderly individuals with severe disability in the rural area were more likely to die because of the aforementioned reasons. In the present study, we identified that disability increased the 4-years mortality and cognitive impairment was associated with male mortality, which is in accordance with the results of previous studies on the oldest-old populations^4–6,9^. In the Danish 1905 cohort study on nonagenarians, Nybo^4^ *et al*. reported that disability and cognitive impairment were both predictors of mortality, with an HR of 1.61 and 1.42, respectively. The author also hypothesized the age-related loss of muscle mass in the oldest-old population might interact with other geriatric syndromes, including disability and cognitive impairment. Previous studies also reported these geriatric syndromes were mediated through similar patho-etiological factors, such as chronic inflammation, insulin resistance, and hormonal disorder. However, in present study, no significant interaction between low muscle mass and severe cognitive impairment or disability was observed. Our data suggested that public health professionals and clinicians of the oldest-old population do not need to consider the effect of deficit accumulation when these geriatric syndromes are taken into account alone.

Our study has several limitations. First, we estimated muscle mass with anthropometric equation, rather than bioelectrical impedance analysis (BIA) or dual-energy X-ray absorptiometry (DEXA) as recommended by the AWGS. Considering the unavailability of DEXA or BIA in Chinese rural area in 2009, and extreme fatigue of the oldest-old participants, anthropometric measure was the most suitable option in our study. Moreover, due to the shortage of equation for the oldest-old population, we had to use an equation derived from Chinese of 18–69 years, which might not have been adequate to estimate the muscle mass in our population. Furthermore, Yang *et al*. recently demonstrated that sarcopenia assessed with the same equations was valuable in the prediction of long-term mortality in Chinese elderly inpatients^38^, which increased our confidence in the reliability of anthropometric measure. In the future, prospective studies which measure muscle mass with more reliable methods (BIA or DEXA) should be conducted to validate our findings. Second, we didn’t measure muscle strength and physical performance due to the high prevalence of disability and immobility, which might be important confounders. Third, according to our definition of low muscle mass, only 32 men (13.4%) met the cutoff value. The small sample will make the analyses extremely unstable and at risk of being unreliable. Forth, we didn’t collected the information on cause of death, which hindered estimating what type of death was mostly associated with the low muscle mass. The lack of information on the cause of death is due to the fact that several elderly individuals chose to die at home instead in the hospital because of Chinese culture, which hindered the accurate recording of the cause of death by the civil affairs department. Fifth, disability was only measured using the self-reported Katz index, and the objective tests of physical performance, such as gait speed, timed up, and go test or five times sit to stand test, were not conducted. Both subjective and objective-tested methods should be included in future studies to assess disability. Lastly, the participants included in present study were over 90 years, and the epidemiology of the oldest-old populations might be different compared to that of the young-old populations. Thus, our results may be used with caution and may not be arbitrarily applicable to other age populations.

## Conclusion

The muscle mass of nonagenarians and centenarians was 6.11 ± 0.53 kg/m^2^ in men and 4.00 ± 0.63 kg/m^2^ in women, respectively. In an adjusted model, low muscle mass was valuable in the prediction of long-term mortality in female nonagenarians and centenarians. In addition, disability and cognitive impairment were also considered as strong predictors. We expect that the public health workers or clinicians should focus on these predictors to improve the survival or life qualify of the oldest-old population.

## Data Availability

Supporting data will be made available on request.
